# Destabilized SMC5/6 complex leads to chromosome breakage syndrome with severe lung disease

**DOI:** 10.1172/JCI82890

**Published:** 2016-07-18

**Authors:** Saskia N. van der Crabben, Marije P. Hennus, Grant A. McGregor, Deborah I. Ritter, Sandesh C.S. Nagamani, Owen S. Wells, Magdalena Harakalova, Ivan K. Chinn, Aaron Alt, Lucie Vondrova, Ron Hochstenbach, Joris M. van Montfrans, Suzanne W. Terheggen-Lagro, Stef van Lieshout, Markus J. van Roosmalen, Ivo Renkens, Karen Duran, Isaac J. Nijman, Wigard P. Kloosterman, Eric Hennekam, Jordan S. Orange, Peter M. van Hasselt, David A. Wheeler, Jan J. Palecek, Alan R. Lehmann, Antony W. Oliver, Laurence H. Pearl, Sharon E. Plon, Johanne M. Murray, Gijs van Haaften

**Affiliations:** 1 Department of Genetics (Center for Molecular Medicine) and; 2 Department of Pediatric Intensive Care, Wilhelmina Children’s Hospital, University Medical Center Utrecht (UMCU), Utrecht, Netherlands.; 3 Genome Damage and Stability Centre, School of Life Sciences, University of Sussex, Falmer, United Kingdom.; 4 Human Genome Sequencing Center,; 5 Department of Molecular and Human Genetics,; 6 Texas Children’s Hospital, and; 7 Department of Pediatrics, Baylor College of Medicine, Houston Texas, USA.; 8 Central European Institute of Technology and Faculty of Science, Masaryk University, Brno, Czech Republic.; 9 Department of Pediatric Immunology and Infectious Diseases,; 10 Department of Pediatric Pulmonary Diseases, and; 11 Department of Metabolic Diseases, Wilhelmina Children’s Hospital, UMCU, Utrecht, Netherlands.

## Abstract

The structural maintenance of chromosomes (SMC) family of proteins supports mitotic proliferation, meiosis, and DNA repair to control genomic stability. Impairments in chromosome maintenance are linked to rare chromosome breakage disorders. Here, we have identified a chromosome breakage syndrome associated with severe lung disease in early childhood. Four children from two unrelated kindreds died of severe pulmonary disease during infancy following viral pneumonia with evidence of combined T and B cell immunodeficiency. Whole exome sequencing revealed biallelic missense mutations in the *NSMCE3* (also known as *NDNL2*) gene, which encodes a subunit of the SMC5/6 complex that is essential for DNA damage response and chromosome segregation. The *NSMCE3* mutations disrupted interactions within the SMC5/6 complex, leading to destabilization of the complex. Patient cells showed chromosome rearrangements, micronuclei, sensitivity to replication stress and DNA damage, and defective homologous recombination. This work associates missense mutations in *NSMCE3* with an autosomal recessive chromosome breakage syndrome that leads to defective T and B cell function and acute respiratory distress syndrome in early childhood.

## Introduction

Chromosome dynamics in eukaryotes are controlled by the structural maintenance of the chromosome complex (SMC) family of proteins, which form 3 highly conserved and functional complexes: cohesin (SMC1/SMC3), condensin (SMC2/SMC4), and SMC5/6 (1). The SMC5/6 complex, consisting of SMC5, SMC6, and non-SMC elements NSMCE1–6, has key roles in the maintenance of chromosome integrity during mitotic proliferation, meiosis, and DNA repair and is critical for genome stability (2–4). In particular, the SMC5/6 complex is involved in resolving intermediates during recombination (5, 6) and other complex DNA structures, such as stalled replication forks (7–9).

Recently, patients with primordial dwarfism, extreme insulin resistance, gonadal failure, and signs of mild spontaneous chromosome instability were identified as carrying mutations in *NSMCE2*, which encodes the SUMO ligase subunit of the SMC5/6 complex (10). Here, we report on patients with very different clinical features carrying mutations in *NSMCE3* (also known as *NDNL2* and *MAGEG1*; OMIM *608243) (11–13), encoding another SMC5/6 subunit, a member of the melanoma-associated antigen (MAGE) protein family (14, 15). Homozygous and compound heterozygous mutations were found in 2 independent kindreds, in which 4 children died in early childhood after developing rapidly progressive pulmonary disease following viral pneumonia. The presence of multiple de novo chromosome rearrangements and variable numbers of de novo supernumerary marker chromosomes in peripheral blood lymphocytes during critical illness together with combined T and B cell immunodeficiency seen in these patients indicates an essential role for the SMC5/6 complex for normal human lymphocyte development and function. These findings extend the spectrum of the previously known chromosome breakage syndromes.

## Results

**Figure 1:**
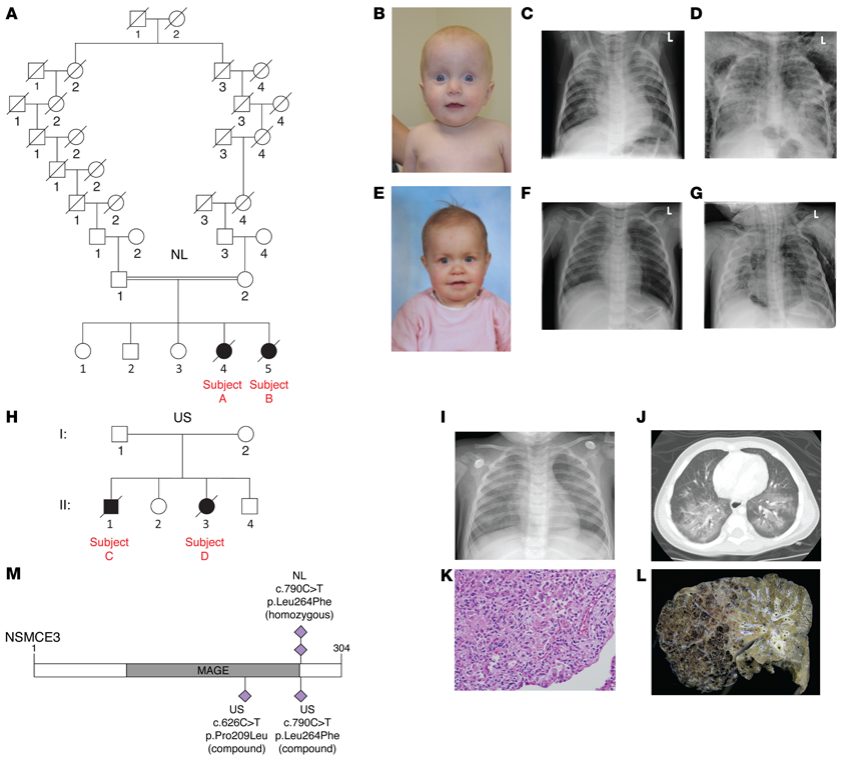
Affected individuals with *NSMCE3* mutations with severe lung disease immunodeficiency and chromosome breakage syndrome (LICS). (**A**) Pedigree of family 1. (**B**) Facial appearance of affected individual A (note the thin skin and prominent veins). (**C**) Chest X-ray of individual A, 4 days before admission at the PICU, showing severe PARDS consisting of bilateral alveolar infiltrates and (**D**) 14 days after admission, showing diffuse interstitial and alveolar infiltrates, pneumomediastinum, and subcutaneous emphysema. (**E**) Facial appearance of affected individual B. No dysmorphic facial features were noted. (**F**) Chest X-ray of individual B on admission at the PICU showing a predominantly right-sided alveolar infiltrate and (**G**) 18 days after admission showing severe PARDS complicated by pneumomediastinum, pneumothorax, and subcutaneous emphysema. (**H**) Pedigree of family 2. (**I**) Chest X-ray showing bilateral interstitial infiltrates of affected individuals C and D. (**J**) Corresponding chest CT scan at the level of the carina showing bilateral ground glass haziness with areas of consolidation and interposed air bronchogram. (**K**) Lung biopsy of individual D at day 6 showing patchy acute interstitial infiltrates with lymphocyte predominance. In the areas of parenchymal injury, there was marked alveolar epithelial hyperplasia, consistent with early diffuse alveolar damage (original magnification, ×400). (**L**) Lung explant showing significant damage that includes overinflation, macroscopic cystic changes, and intracystic hemorrhage. (**M**) Schematic representation of the NSMCE3 protein with the identified missense mutations of the affected individuals. The homozygous mutations from the affected individuals from the Netherlands (NL; A and B) are indicated above and the compound heterozygous mutations from the affected individuals from the United States (US; C and D) are depicted below the figure.

The probands of the first kindred were 2 sisters from distantly related, healthy Dutch parents of European descent (Figure 1A, Table 1, Supplemental Figure 1, and Supplemental Data, Family 1; supplemental material available online with this article; doi:10.1172/JCI82890DS1). The affected individuals (referred to as affected individual A [GVH01] and B [GVH02]) died at the ages of 12 and 14 months, respectively, due to severe progressive irreversible lung damage following multiple virus-induced pneumonia episodes. After onset of pediatric acute respiratory distress syndrome (PARDS) (16), their pulmonary status was complicated by pneumomediastinum, pneumothorax, and subcutaneous emphysema (Figure 1, C, D, F, and G).

Prior to these episodes, the children experienced feeding difficulties, failure to thrive, weight loss, mild psychomotor retardation, axial hypotonia, increased infection susceptibility (due to distinct B and T cell abnormalities), and eczema. Only mild or no dysmorphic features were noted (Figure 1, B and E, and Table 1). Karyotyping of cultured peripheral lymphocytes drawn either before (individual A) or during illness (individual B) revealed multiple de novo supernumerary marker chromosomes with or without additional de novo chromosome rearrangements (Supplemental Figure 2). Array comparative genomic hybridization (aCGH) analysis of genomic DNA from blood did not show chromosome gains or losses, indicating the stochastic nature of the imbalances. Immunological analysis in individuals A and B showed that the numbers of T lymphocytes were low, but B lymphocytes and immunoglobulins were normal (except for increased IgM). Several specific antibody responses after vaccination were low, and T lymphocyte proliferation after stimulation with recall antigens was absent or decreased (Table 2 and Supplemental Data, Family 1).

The unprecedented clinical and cytogenetic features observed in the patients prompted us to perform whole exome sequencing in this family. Sequencing of individuals A and B, parents, and an unaffected sibling yielded 110,896 non-reference positions found in at least one of the family members. Crosschecks with available databases revealed 63,734 variants not present in either dbSNP (http://www.ncbi.nlm.nih.gov/SNP/, build 134, August 2011) or our in-house database of 150 exomes. Homozygosity mapping and genealogic analysis showed evidence of distant-relatedness of the parents (see Methods). The largest homozygous region spanned 8.4 Mb (chr15:25,144,362-33,537,514 [GRCH38/hg38]; 15q11.2–q13.3), and within this region one single, rare, and likely pathogenic variant was found: a homozygous G-to-A substitution at chr15:29268916 in the coding region of *NSMCE3*. Sanger sequencing confirmed the homozygosity of variants in affected individuals and heterozygosity in the tested family members. The likely pathogenic variant results in a p.Leu264Phe missense change in NSMCE3 (NM_138704.3:c.790C>T) in a conserved region of the protein.

The c.790C>T variant has been detected in the heterozygous state among 662 individuals by the CLINSEQ project and annotated as rs199905054 (17, 18). In the Genome of the Netherlands (GoNL) study, the variant has been observed in the heterozygous state 3 times in 500 unrelated individuals, indicating an allele frequency of 0.003 in the Netherlands (19), showing that the variant is not uncommon in the Dutch population. The heterozygous allele frequencies in the European and global population are 4.5 × 10^–5^ and 2.5 × 10^–5^, respectively (Exome Aggregation Consortium [ExAC]; http://exac.broadinstitute.org, August 2015), with no homozygous individuals.

In a search of *NSMCE3* using GeneMatcher (https://genematcher.org/) (20), we identified a second family with a remarkably similar clinical history and research whole exome sequencing data in the United States. This family included an affected brother and sister (respectively, individual C [11179-05] and individual D [FCP488]), of healthy, unrelated parents of European descent [FCP645 and FCP646]) (Figure 1H, Table 1, and Supplemental Data, Family 2). Individual C presented with failure to thrive at 5 months of age, for which no apparent cause could be identified. At 14 months of age, he developed progressive, severe PARDS following infectious pneumonia that led to his death. Genetics consultation included a peripheral blood karyotype and multiple metabolic analyses without a diagnosis (Supplemental Data, Family 2). The autopsy findings revealed acute eosinophilic pneumonia, diffuse alveolar damage, extensive lobular fibrosis, bronchiolitis obliterans, and organizing pneumonia. Individual D, who also presented with significant growth restriction, developed severe pneumonitis at the age of 13 months and needed prolonged mechanical ventilation (Figure 1, I and J). Lung biopsy showed patchy acute interstitial infiltrates with lymphocyte predominance (Figure 1K). She underwent cadaveric lung transplantation at 15 months of age because of severe pulmonary damage initially resembling PARDS. The histopathology of her explanted lungs revealed end-stage lung disease due to organizing pneumonia with extensive lobular remodeling, obliteration of air spaces, interstitial fibroplasia, cystic remodeling and marked hyperinflation, and patchy interstitial hemorrhage (Figure 1L). Within the following year, she developed bone marrow failure and increased infection susceptibility, including sternal osteomyelitis. Her clinical status deteriorated, and individual D died at 32 months of age. Peripheral blood karyotype in lymphocytes of individual C at 6 months of age and when not acutely ill showed a normal (46,XY) karyotype, and in individual D breaks at 3p13~14 were identified in 2 of 28 examined cells. Metaphase spreads from 100 early-passage fibroblasts from individual D showed stochastic chromosome rearrangements in 13 cells (Supplemental Figure 2). Both affected individuals, C and D, showed diminished T cell percentage and an increased B cell percentage around 13 months of age (Table 2). A clinical radiosensitivity assay of lymphocytes (21) from individual D demonstrated increased sensitivity to 1 Gy of ionizing radiation (IR; 16% survival with normal range 50% ± 13%), similar to cells from patients with ataxia telangiectasia (AT) (14% ± 17%). Western blot analysis showed a 48% reduction in ATM protein compared with the 85% reduction seen in patients with AT.

Research exome sequencing of individual D and both parents revealed 24,500 germline variants. Because of the suspected autosomal recessive inheritance pattern, variants were filtered according to homozygosity and compound heterozygosity. No rare or novel homozygous coding variants (segregating in the parents) were identified. Two genes with rare compound heterozygous coding variants were identified: *INPP5J* and *NSMCE3*. However, both missense variants in *INPP5J* were predicted to be benign by Polymorphism Phenotyping v2 (PolyPhen-2; http://genetics.bwh.harvard.edu/pph2/), and no rare variants were observed in *INPP5J* in the Dutch family (individuals A and B), despite good coverage. Two missense and likely pathogenic variants in *NSMCE3* were found (Figure 1M), consisting of a maternally inherited p.Leu264Phe (NM_138704.3:c.790C>T) change (identical to individuals A and B) and a paternally derived G-to-A substitution resulting in a p.Pro209Leu change. Both variants were confirmed by Sanger sequencing. Directed Sanger analysis of DNA from lung tissue from the sibling, individual C, demonstrated both alleles. The p.Pro209Leu mutation was predicted “possibly damaging” by PolyPhen-2 (0.752) and deleterious by SIFT (0.03; http://sift.jcvi.org/) and is absent in the ExAC database, despite good coverage at that position.

Our genetic studies of 2 unrelated kindreds with remarkably similar clinical histories provide a strong link between missense mutations in *NSMCE3* and the phenotypes of chromosomal instability and failure to thrive with infectious (viral) pneumonia ending in fatal lung disease. Together with the rarity of the variants and the predicted damaging effect at the protein level, these data suggest the identification of a chromosome breakage disease due to alteration of the *NSMCE3* component of the SMC5/6 complex, which is essential for responses to DNA damage and chromosome segregation during cell division.

### NSMCE3 mutant proteins destabilize the SMC5/6 complex.

**Figure 2:**
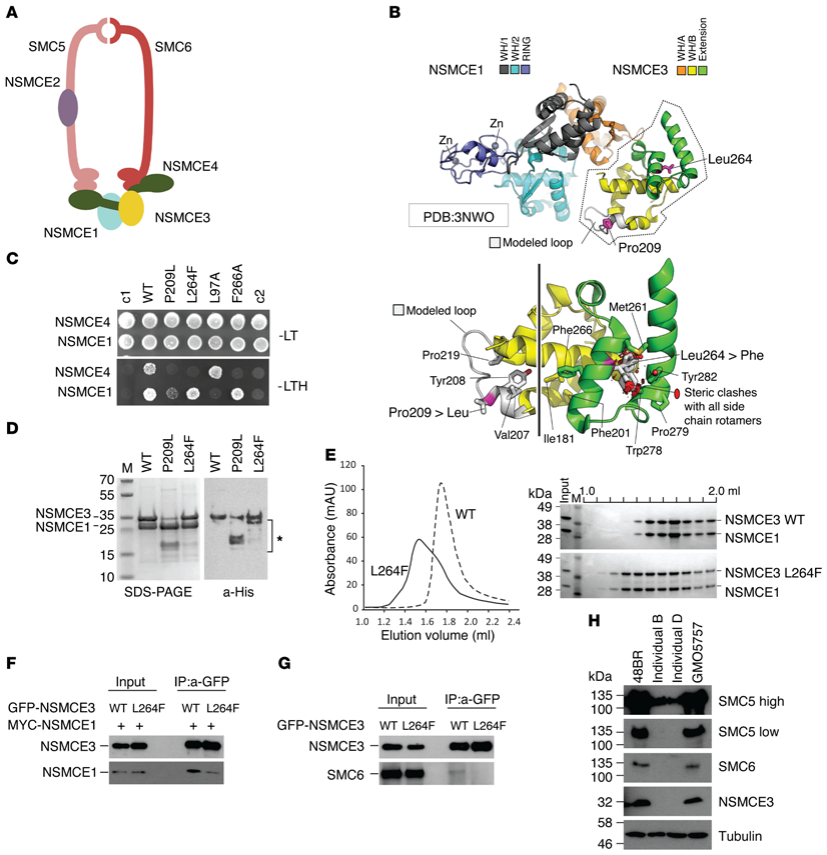
*NSMCE3* mutations destabilize the SMC5/6 complex. (**A**) Schematic of the SMC5/6 complex. (**B**) Structure of NSMCE1/NSMCE3, highlighting the positions of the Leu264Phe (L264F) and Pro209Leu (P209L) mutants that destabilize the WH/B subdomain of NSMCE3. Loops missing from the deposited structure were modelled and are shown in gray (24). (**C**) Yeast 2-hybrid analysis showing interactions between NSMCE3 and NSMCE1 or NSMCE4, scored on His^–^ plates containing 5 mM 3-amino-1,2,4-triazol (-LTH). WT denotes WT NSMCE3; L97A: NSMCE3 Leu97Ala, which abrogates NSMCE1 binding (25); F266A: NSMCE3 Phe266Ala, which reduces binding to NSMCE4 (21). L264F only interacts with NSMCE1; P209L has reduced interaction with NSMCE1 and no interaction with NSMCE4. Controls: c1, BD-NSMCE3 with empty AD vector; c2, empty BD vector with AD-NSMCE4 (top) or NSMCE1 (bottom). (**D**) NSMCE1 and His-tagged NSMCE3 (WT, P209L, and L264F were coexpressed in, and purified from, *E*. *coli*. Left: Coomassie-stained SDS-PAGE shows copurification of NSMCE1 and NSMCE3. Right: Immunodetection of His-tagged NSMCE3. P209L is unstable; L264F is more stable. (**E**) Size exclusion chromatography column. NSMCE1 and NSMCE3 co-elute, forming a stable complex. NSMCE1 and L264F co-elute, over a broader range of volume and non-uniform distribution, indicating either unfolding of L264F or interaction with the chromatography resin. (**F**) GFP-tagged NSMCE3 (WT or L264F) and Myc-tagged NSMCE1 were ectopically expressed in human U20S osteosarcoma cells; proteins were stable and full length in input samples. Proteins were immunopurified using anti-GFP beads. Purification of NSMCE1 was reduced when copurified with L264F, which indicated reduced affinity. (**G**) GFP-tagged NSMCE3 (WT or L264F) was ectopically expressed in U20S cells. Proteins were immunopurified using anti-GFP beads. Reduced copurification indicates a compromised integration of the mutated NSMCE3 into the SMC5/6 complex. (**H**) Western blot analysis of SMC5/6. NSMCE3 is undetectable in fibroblasts from individual B (GVH02) and individual D (FCP488) compared with primary fibroblast cell lines (48BR and GM05757). High exposure of anti-SMC5 blot shows reduction of SMC5. Levels of both SMC5 and SM6 were reduced in patient fibroblasts, indicating destabilization of the complex. Loading control: tubulin.

Within the SMC5/6 complex the interaction of NSMCE1, NSMCE3, and NSMCE4 is essential for the formation of a tight subcomplex (22–24), which bridges the large SMC5 and SMC6 subunits (refs. 23, 24, and Figure 2A). NSMCE3 interacts with NSMCE1 through its N-terminal winged-helix (WH) domain, WH/A, and with NSMCE4 via its C-terminal WH domain, WH/B (22–24). We studied the effect of the 2 variants, p.Pro209Leu and p.Leu264Phe, found in the 4 affected individuals on NSMCE3 interactions within the SMC5/6 protein complex. p.Pro209Leu is found within the C-terminal WH/B and p.Leu264Phe in the WH/B “extension” (WH/B-e) subdomains of the protein (Figure 2B). The destabilizing effect of both mutations is predicted by computational analyses of models based on the crystal structure of the NSMCE1-NSMCE3 dimer (Protein Data Bank [PDB] ID 3NW0; http://www.rcsb.org) (ref. 25 and Figure 2B). Leu264Phe is predicted to disrupt fold in the NSMCE3 WH/B-e domain because of steric clashes with side chains on the adjacent helix, whereas Pro209 ends the preceding helical element, so the Pro209Leu mutation is likely to disrupt the secondary structure of the protein.

To determine the effect of the patient mutations on the interactions between NSMCE3 and NSMCE1 or NSMCE4, we first used yeast 2-hybrid studies to compare the Leu264Phe and Pro209Leu missense mutations with 2 previously characterized *NSMCE3* mutants (Leu97Ala and Phe266Ala) as controls. Leu97Ala structurally perturbs the WH/A subdomain of NSMCE3, which interacts with NSMCE1 (26), whereas Phe266Ala perturbs the WH/B subdomain, which interacts with NSCME4 (22). The Leu264Phe mutation of the affected individuals A–D abolished binding of NSMCE3 to NSMCE4, while its interaction with NSMCE1 was only mildly affected. The Pro209Leu mutation of the affected individuals C and D, as well as abolishing the NSMCE4 interaction, also drastically reduced the NSMCE1 interaction, confirming that both missense changes directly affect the structure of the WH/B and WH/B-e subdomains (Figure 2C).

Next we purified recombinant human NSMCE1 and NSMCE3 proteins coexpressed in *E*. *coli*. Both the WT and Leu264Phe forms of NSMCE3 co-purified in a roughly 1:1 stoichiometric ratio with (untagged) NSCME1. The Pro209Leu variant, however, proved to be highly prone to C-terminal truncation (as judged by Western blot analysis), resulting in a species migrating on SDS-PAGE gels with a smaller molecular mass. This species corresponded to just the N-terminal WH/A subdomain of NSMCE3 (Figure 2D), and this variant was not analyzed further. Size-exclusion chromatography using the purified recombinant proteins showed NSMCE1 and NSMCE3 to form a stable dimer. The Leu264Phe variant still interacts and co-elutes with NSMCE1, but elutes over a much broader volume and with a non-uniform distribution (Figure 2E). It is likely that unfolding of the WH/B-e subdomain promotes direct nonspecific interactions with the chromatography resin.

To investigate the stability of the Leu264Phe variant in human cells, we coexpressed WT NSMCE3 and the Leu264Phe NSMCE3 variant with NSMCE1 in human U2OS cells. Both WT and variant NSMCE3 proteins were expressed to similar levels and were stable. Coimmunoprecipitation showed that the interaction between NSMCE1 and the variant NSMCE3 Leu264Phe was slightly reduced (Figure 2F). Consistent with the destabilization of the complex seen in patient cells, the Leu264Phe variant showed reduced incorporation into the native SMC5/6 complex (Figure 2G).

Finally, we investigated the effect of the weakened interactions of the mutant NSMCE3 proteins on the endogenous SMC5/6 complex in primary fibroblasts of 1 affected individual from each kindred (individuals B and D). NSMCE3 levels in both patient fibroblasts were dramatically reduced to levels that were below the level of detection in our assay. In addition to NSMCE3, levels of the complex subunits SMC5 and SMC6 were also significantly reduced, indicating that the stability of the SMC5/6 protein complex is compromised in affected individuals of both kindreds (Figure 2H). Together these data show that the identified missense variants in *NSMCE3* destabilize NSMCE3 and the SMC5/6 complex.

### Cells of affected individuals exhibit increased cellular sensitivity to genotoxins and defective homologous recombination.

**Figure 3:**
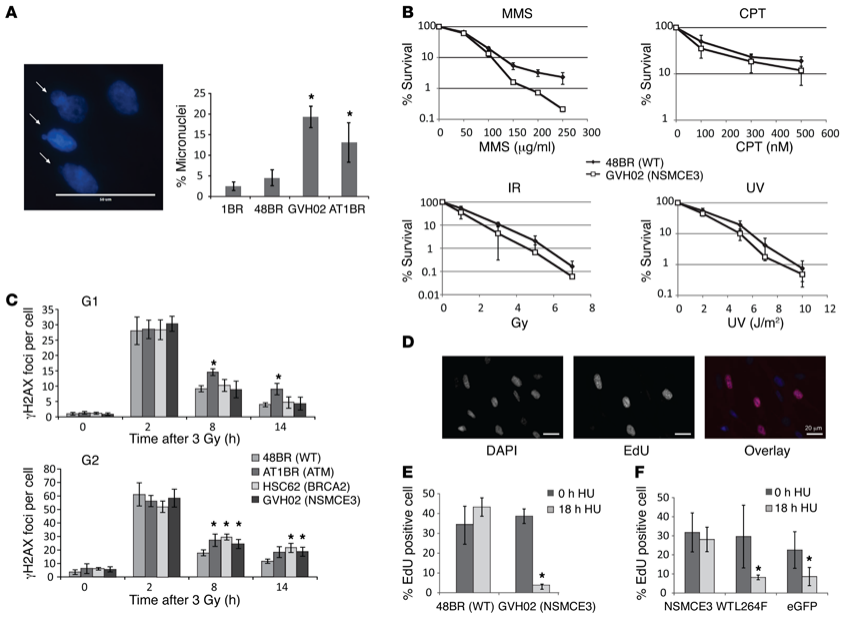
Cellular defects resulting from destabilization of SMC5/6. (**A**) Example of micronuclei (indicated by arrows) and quantification of percentage of cells with micronuclei in fibroblasts from individual B (GVH02), control fibroblasts (1BR and 48BR), and an individual with ATM (AT1BR). Scale bar: 50 μm. (**B**) Survival of primary fibroblasts from individual B (GVH02) compared with the control 48BR fibroblasts after treatment with MMS, IR, CPT and UV light shows a modest sensitivity to all agents. (**C**) Analysis of γH2AX foci after IR in either G_1_ or G_2_. Control (48BR), affected individual B (GVH02), ATM (AT1BR), and BRCA2 (HSC62) fibroblasts were irradiated with 3 Gy IR and incubated for the designated times before fixing and mounting. *ATM^–/–^* cells showed a defect in the slow repair fraction in both G_1_ and G_2_. Cells of individual B, like HR-defective BRCA2 cells, show a defect in G_2_. (**D**) Representative example of EdU-positive cells scored in **E**. Left: DAPI; middle: EdU; right: merge. Scale bars: 20 μm. (**E**) HU recovery assay. Normal (48BR) or individual B’s (GVH02) fibroblasts were incubated for 18 hours either with or without 250 μM HU. HU was removed, and the number of cells incorporating EdU (thymidine analog) in the following 30 minutes was analyzed. (**F**) Complementation of the HU recovery defect. Individual B’s (GVH02) primary fibroblasts were transfected with GFP-tagged normal (NSMCE3 WT) or Leu264Phe variant NSMCE3 (NSMCE3 L264F) cDNA or empty vector (eGFP). The cells were treated with or without HU after 48 hours, and the number of transfected cells (identified by GFP fluorescence) that incorporated EdU was assessed. Results shown in **A**–**C** and **E** and **F** represent mean ± SD of 3 experiments. *P < 0.05, Student’s *t* test, 2-tailed.

Studies in a range of organisms have shown that mutations in components of the SMC5/6 complex confer genome instability, and sensitivity to DNA damaging agents and particularly to replication stress (7, 27–29). Early-passage primary fibroblasts from individual B (homozygous for the Leu264Phe variant) exhibited significantly increased levels of micronuclei, a finding that indicates genome instability and is consistent with increased chromosome breakage. Approximately 20% of cells contained micronuclei, which is similar to levels seen in ATM fibroblasts (Figure 3A) and to cells with mutations in *NSMCE2*, which encodes another component of the SMC5/6 complex (10).

We exposed fibroblasts from individual B to a variety of damaging agents and compared their sensitivities to those of normal cells (Figure 3B). We found modest, but reproducible, hypersensitivity to different agents that produce different types of DNA damage (double-strand breaks [DSBs], methylated bases, topoisomerase-induced breaks and base damage, respectively). Analysis of γH2AX foci, a surrogate marker for DNA damage, showed that repair kinetics in G_1_ cells were unaffected, consistent with proficient nonhomologous end joining (NHEJ). However, similar to homologous recombination–defective (HR-defective) *BRCA2^–/–^* cells, individual B’s fibroblasts had a defect in the slow repair fraction in G_2_ (Figure 3C), consistent with a defect in HR (30). HR is required to stabilize stalled forks and rescue replication (31). Consistently, in multiple experiments, the most dramatically altered response was seen in response to hydroxyurea (HU), a DNA replication inhibitor. After release of the HU block, DNA replication recovered rapidly and completely in normal fibroblasts, as evidenced by incorporation of 5-ethynyl-2′-deoxyuridine (EdU), but not in the fibroblasts of the affected individual (Figure 3, D and E), even though similar levels of cells were arrested in S phase (Supplemental Figure 3). This defect in recovery from replication stress is similar to that in *NSMCE2*-defective cells (10). The defect was restored by expression of normal NSMCE3 protein but not with the mutant protein (p.Leu264Phe), thus confirming that the replication recovery defect results from the *NSMCE3* mutation (Figure 3F).

## Discussion

Here, we identify homozygous and compound heterozygous missense mutations in *NSMCE3* as the cause of what we believe to be a new autosomal recessive chromosome breakage syndrome, characterized by failure to thrive, absent (or mild) dysmorphic features, immune deficiency, and severe and eventually fatal pediatric pulmonary disease, initially resembling PARDS, in early childhood. The proposed disease name is lung disease immunodeficiency and chromosome breakage syndrome (LICS). Lymphocytes of affected individuals showed increased chromosomal breakage, and fibroblasts were sensitive to DNA-damaging agents. The underlying lymphocyte proliferation defect is consistent with the destabilization of the SMC5/6 complex seen in fibroblasts and consequential defect in HR (32).

Both of the *NSMCE3* missense mutations identified in these kindreds destabilize NSMCE3. Levels of NSMCE3 were below detection levels in fibroblasts from affected individuals B (homozygous p.Leu264Phe) and D (compound heterozygote p.Leu264Phe, p.Pro209Leu). This could be due to lack of expression of NSMCE3, but, since loss of *SMC6* or *NSMCE2* is lethal in early embryonic mice (33, 34), it is likely that NSMCE3 is present at low levels in the patient cells but below the detection level of our antibodies. The levels of SMC5 and SMC6 were also significantly reduced, showing the complex to be destabilized. In vitro analysis suggests that Pro209Leu leads to C-terminal truncation of NSMCE3. In contrast, the Leu264Phe variant, while still forming a complex with NSMCE1, has a reduced interaction with NSMCE4 and SMC6. The destabilization of the SMC5/6 complex is much more severe than the mild effect seen in cells from individuals with a truncating mutation in *NSMCE2*, encoding the SUMO ligase subunit of SMC5/6 (10). Since NSMCE2 SUMO ligase activity is not required for all SMC5/6 functions (34), this may explain the differences in clinical features. As both mutations in *NSMCE3* disrupt the interaction with NSMCE4, we speculate that such mutations specifically result in the pulmonary phenotype that we have observed. Additional phenotype and genotype data are necessary to further evaluate this hypothesis.

In cells from affected individual B, the destabilization of the SMC5/6 complex resulted in defective DNA repair by HR but not by NHEJ. This is consistent with previous findings in the literature (4, 6, 7, 28, 35). Consistent with the HR defect, fibroblasts of affected individual B had a defect in the recovery from replication stress, which could be complemented by expression of WT NSMCE3 but not p.Leu264Phe, showing the defect to be a direct consequence of the mutation. The lack of recovery from replication stress is also seen in cells mutated in *NSMCE2* (10), showing it to be a common consequence of misregulation of the SMC5/6 complex.

The affected individuals share some clinical features with individuals with Nijmegen breakage syndrome (NBS) and AT chromosomal breakage syndrome (Tables 1 and 2). In NBS, the affected protein, nibrin, along with MRE11 and RAD50, forms the MRN complex, which is required for DSB repair (both NHEJ and HR) (36) and together with ATM, the protein mutated in AT, is necessary for proper ATM-mediated DNA damage signaling response to DSBs (36, 37).

In the syndrome associated with mutations in *NSMCE2*, linear growth, weight, and head circumference growth were severely impaired (10). In the affected individuals described here, linear growth and weight were slightly affected in individuals A and B, but more severely affected in individuals C and D. This difference might be explained by the more severe effect of p.Pro209Leu, present in the latter 2 affected individuals. Deregulation of glucose metabolism, observed in AT as well as in the *NSMCE2*-associated syndrome (10), was not noted in the affected individuals described here prior to their illness. During their stay at the pediatric intensive care unit (PICU), individuals A and B had (slightly) elevated glucose plasma levels, a feature commonly seen during critical illness (38). No insulin plasma levels were measured to correlate with glucose levels to provide further insight into the possible presence of insulin resistance. No abnormalities in glucose regulation have been noted in individuals C and D.

No malignancies were reported in these 4 affected individuals, including autopsy analysis of individuals A, C, and D, in contrast to patients with AT and NBS. However, the death of all 4 children in very early childhood would obscure any predisposition to cancer. Bone marrow failure was reported in individual D after she received a lung transplant, shortly before death.

The increased susceptibility to specific infections and specific allergens in the 4 affected individuals have also been observed in AT and NBS (39). Common respiratory tract viruses were isolated at the beginning, but no longer found during follow-up in individual B, indicating intactness of at least part of the cytotoxic immune response. This is consistent with the laboratory evaluations excluding SCID, but instead pointing to a distinct, combined T and B cell immunodeficiency. The fact that in the 4 affected individuals B cell counts were normal indicates proper efflux from the bone marrow, which is defective in AT and NBS (40, 41). This normal efflux would fit with the normal NHEJ, and hence normal V(D)J recombination, shown in the cells from the subjects reported here.

The poor antibody responses to specific vaccinations as well the decreased recall antigen proliferation are also seen in NBS (40). This effect has previously been attributed both to an absolute decrease in the number of B lymphocytes and to a reduced proportion of switched memory B lymphocytes secondary to defective V(D)J and class switch recombination (CSR) (40), respectively. In patients with AT, the disturbed naive B cell and T cell homeostasis has been related to reduced B and T cell production caused by disturbed V(D)J recombination. This defect also leads to a limited B cell and T cell receptor repertoire (41). In the 4 affected individuals described here, B lymphocyte levels were normal, and no signs of a defective V(D)J recombination defect were present.

Although it has previously been shown that HR is necessary for normal B cell lymphocyte development (32), data from our patients suggest an important role for HR in normal T cell development. The decreased T cell counts could alternatively be indicative of increased apoptosis or defective thymic output, which could well fit the thymic hypo- and aplasia observed in patients A and D and which had also been described in AT (41) and NBS (42).

Despite extensive attempts by investigators in both the United States and European centers, we were unable to identify clinically similar patients prior to the identification of these 2 kindreds by the use of GeneMatcher. This highlights the importance of data sharing through GeneMatcher and other avenues being developed by the Matchmaker Exchange of the Global Alliance for Genomics and Health (43). However, the carrier incidence, especially in the Netherlands, would predict the existence of several other cases. The fact that no other patients were identified presumes that, so far, these patients have remained undiagnosed. For the 2 families reported here, suspicion of an underlying chromosome instability syndrome occurred only after a similar disease history was noted in a second sibling. Prior to the rapid onset of fatal pulmonary disease, clinical symptoms of individuals affected by this breakage syndrome were relatively mild and could not be classified. Clinical assays, such as karyotyping and IR sensitivity, could be abnormal and can be early indicators of the syndrome. We recommend development of a clinical assay for the replication recovery defect and for *NSMCE3* mutations and consideration of this disorder for children with unexplained, rapid pulmonary failure following multiple virus-induced pneumonias.

In summary, our work describes what we believe to be a novel autosomal recessive chromosome breakage syndrome, resulting from missense mutations in *NSMCE3*. These mutations affect the stability of the SMC5/6 complex and HR, leading to defects in the function of T and B cell lymphocytes. This disorder, designated LICS, which expands the spectrum of chromosomal breakage syndromes, is associated with progressive, severe PARDS following viral pneumonia, resulting in death in early childhood of the affected individuals described here.

## Methods

### Research subjects.

Genomic DNA from the affected individuals and family members was extracted from peripheral blood using standard methods.

### Genealogy and pedigree construction, family 1.

Birth, marriage, and death records, personal cards of the Dutch civil registration (years 1811–2013), and church books (before 1811) were used for pedigree construction.

### Blood and fibroblast samples.

The blood samples used for karyotyping in lymphocytes of individual A were drawn at the age of 9 months and at the age of 12.5 months, when she presented with severe pulmonary insufficiency, during her stay at the PICU. The cultures of dermal fibroblasts were taken from individual A (at the beginning and the end) at the PICU only. The blood sample used for karyotyping in lymphocytes and the culture for dermal fibroblasts from individual B were taken during her stay at the PICU only at the age of 14 months. The blood sample used for karyotyping in lymphocytes of individuals C and D was performed on peripheral blood lymphocytes at the ages of, respectively, 6 months and 2 years and 5 months.

### Genetic and cytogenetic analysis.

Karyotyping of short-term cultures of stimulated peripheral blood lymphocytes and dermal fibroblasts, sequencing of candidate genes, and aCGH (BlueGnome Cytochip v2.0 [individual A] and Agilent 180K oligonucleotide platform [patient B]) based on DNA from blood were all performed in a clinical diagnostic setting according to standard procedures. Exome sequencing was performed as described previously for the Dutch kindred (44) and for the US kindred (45). The variants were deposited to ClinVar (http://www.ncbi.nlm.nih.gov/clinvar/; SCV000266831 and SCV000266832), exome sequencing data within the 8.4-Mb homozygous region of the Dutch kindred were deposited to the European Genome-phenome Archive (https://www.ebi.ac.uk/ega/home; EGAS00001001786). Testing of candidate genes and methylation-specific multiplex ligation-dependent probe amplification (MS-MLPA) were performed in a clinical diagnostic setting. Karyotyping in lymphocytes was performed by G-banding at 500–550 band resolution. Testing of skin fibroblasts for sensitivity to radiation and to mitomycin C followed standard clinical diagnostic procedures. aCGH based on DNA from peripheral blood was performed using either BlueGnome Cytochip v2.0 (individual A) or the Agilent 180K oligonucleotide platform (individual B) following instructions provided by the manufacturer. As a reference DNA sample, a mixture of genomic DNA from 50 normal, healthy females was used.

### Homozygosity mapping.

Genomic DNA samples from both patients in family 1, their parents, and 2 of the unaffected siblings were analyzed using Illumina HumanCytoSNP-12v2 arrays according to the protocol of the manufacturer (Illumina). Regions of homozygosity were determined using BeadStudio software. Windows of at least 20 homozygous SNPs were detected for each tested individual; no mismatches were allowed. Homozygous regions with the same start and end points present in both patients or in any of the unaffected family members were excluded on the basis of the identity by descent (IBD) premise (46). Only regions exclusively present in the affected children or regions overlapping between affected and unaffected family members with different start and end points were considered for further analysis. Regions overlapping gene deserts around centromeres were excluded.

### Micronuclei protocol.

Fibroblasts were grown to approximately 70% confluence, trypsinized, resuspended, counted, and seeded onto glass coverslips. Twenty-four hours later, cells were fixed using 4% paraformaldehyde (PFA) for 10 minutes at room temperature. Cells were then mounted on glass slides using ProLong Gold Antifade Mountant with DAPI (Life Technologies). Approximately 1,000 cells were analyzed over 3 independent experiments.

### Clonogenic survival assays.

Clonogenic survival assays were carried out as described previously by Arlett et al. (47, 48). Cells were plated in triplicate on 10-cm dishes with feeder cells (1BR normal donor fibroblasts) after treatment with indicated doses of DNA-damaging agent (HU: 0, 0.25, 1, 5 mM for 18 hours; camptothecin [CPT]: 0, 100, 300, 500 nM for 1 hour; methyl methanesulfonate [MMS]: 50, 100, 150, 200, 250 μg/ml for 1 hour; IR: 0, 1, 3, 5, 7, 10 Gy; UV: 0, 2, 5, 7, 10 Jm^–2^). Feeder cells were irradiated with 35 Gy γ-rays. After treatment, cells were washed with PBS, supplied with fresh prewarmed media, and incubated at 37°C with 5% CO_2_ for 21 days until the formation of macroscopic colonies. After 21 days, cells were treated with 1 ml 10% methylene blue in 10 ml media. Survival was calculated by dividing the average number of colonies on treated plates by the average number of colonies on untreated plates, correcting for plating efficiencies. Data are the mean ± SD of 3 biological replicates.

### Repair pathway assay.

DNA repair pathway defects were investigated by monitoring the rate of decline in γH2AX foci as a surrogate marker for recovery after IR in G_1_ or G_2_ phases of the cell cycle (30). NHEJ is the major DSB repair pathway in both G_1_ and G_2_, and HR is only required for repair of about 15% of IR-induced DSBs in G_2_. Consistent with this, BRCA2-deficient cells, defective in HR, are only defective in the slow recovery fraction in G_2_ cells, but *ATM^–/–^* cells are defective in both G_1_ and G_2_ (30).

### Cell culture and irradiation.

Primary fibroblasts from 48BR (WT control), GVH02 (NSMCE3-L264F), HSC62 (BRCA2-deficient primary human fibroblasts from a patient with a homozygous mutation in BRCA2; ref. 49), and AT1BR (*ATM^–/–^*) were grown in MEM supplemented with 15% fetal calf serum, 2 mM l-glutamine, 100 IU ml penicillin, and 100 μg/ml streptomycin.

Cells were grown in 35-mm dishes on glass coverslips for 48 hours before being irradiated using a ^137^Cs γ-ray source at a dose rate of 1 Gy per 9 seconds and exposed for 27 seconds. Cells were fixed using 4% PFA, permeabilized with 0.2% Triton X-100, and washed with PBS. Cells were incubated with primary antibodies for 30 minutes and washed 3 times with PBS before 20 minutes of incubation with secondary antibodies. Slides were mounted using ProLong Gold Antifade Mountant (Life Technologies) containing DAPI and cells imaged using an IX73 Olympus microscope, equipped with a Lumencor LED light source, a 60× 1.4NA PlanApo lens (Olympus), and an Orca Flash CMOS camera (Hamamatsu). Images were analyzed using Micro-Manager and ImageJ (NIH) software (50, 51). Scoring of foci was performed until at least 15 G_2_ cells and 15 G_1_ cells were registered per sample. Experiments were performed in triplicate, and error bars represent the SD between 3 independent experiments and between 2 operators. Statistical analysis using Student’s *t* test was performed at critical time points to evaluate the significance of differences in levels of foci. CENPF staining was used to mark G_2_ cells; S phase cells were identified by pan-nuclear γH2AX staining and were excluded from analysis. Primary antibodies were γH2AX (05-636-1, 1:800, Merck Millipore) and CENPF (Ab5, 1:1,000, Abcam). Secondary antibodies were FITC (mouse F0257, 1:200, Sigma-Aldrich) and Cy3 (rabbit C2306, 1:200, Sigma-Aldrich).

### EdU incorporation assay.

Fibroblasts were seeded onto coverslips and allowed to grow for 24 hours. They were then grown for 18 hours with or without 250 μM HU. This was washed out, and cells were grown in the presence of 10 μM EdU for 30 minutes. EdU detection was carried out by “click chemistry” as per the manufacturer’s protocol (C10337, Life Technologies). At least 100 cells were scored in each condition in each of 3 independent experiments.

### Transient transfection protocol.

One microgram pCI-NEO with NSMCE3 EGFP (WT or NSMCE3-L264F) or EGFP vector control was transfected into patient B fibroblasts on 10-cm dishes using 3 μl GeneJuice transfection reagent (Merck Millipore). The culture medium was replaced 12–18 hours after transfection, and cells were allowed to incubate for a further 24 hours before being assayed in the EdU HU recovery experiment. At least 90 cells were scored in each condition in each of 3 independent experiments.

### Western blotting.

Endogenous levels of NSMCE3, SMC5, and SMC6 were detected via immunoblot analysis. Frozen cell pellets from kindred 2 were shipped to the United Kingdom for analysis in parallel with kindred 1. Cells were lysed in lysis buffer (20 mM HEPES pH 7.4, 0.5% NP-40, 40 mM NaCl, 2 mM MgCl_2_, 1× protease inhibitor cocktail [Roche], 1× phosphatase inhibitor cocktail [Roche], 25 U ml^–1^ benzonase [Merck]) for 30 minutes at 4°C and sonicated (30 seconds 30% amplitude using a micro-tip; Sigma-Aldrich). Total protein concentration was determined by Bradford assay, and 38 μg of whole cell extract was loaded per lane, separated by 10% SDS-PAGE gel, and transferred to nitrocellulose membranes. Membranes were blocked in 3% nonfat dry milk, 0.1% Tween-20, and incubated with primary antibodies overnight at 4°C. HRP-conjugated secondary antibodies were incubated for 1 hour at room temperature, and membranes were developed with chemiluminescence (Pierce ECL Western Blotting Substrate, Thermo Fisher Scientific). Rabbit polyclonal anti-SMC5 (1:100; MW: 130 kDa) (52), rabbit polyclonal anti-SMC6 (1:100; MW: 130 kDa) (52), rabbit polyclonal anti-NSMCE3 (MAGEG1) (1:100; MW: 35 kDa) (14) were used to detect SMC5/6 component proteins, and rabbit polyclonal anti-tubulin (1:1,000; MW: 55 kDa) (2144 lot 4, Cell Signaling Technology) was used as a loading control. NSMCE3 (MAGEG1) antibodies were precleared by Western blotting against U2OS cell lysates. These antibodies detect both endogenous and recombinant NSMCE3 overexpressed in U20S cells at 32 kDa and showed endogenous levels to be reduced by siRNA knockdown of NSMCE3 (Supplemental Figure 4). HRP-conjugated secondary antibodies were from Dako (P0217 lot 0086784). Protein levels were quantified using GeneTools software (SYNGENE), and levels were normalized against tubulin loading control.

### Immunoprecipitation.

U20S cells (~1.5 × 10^6^) were transfected with 10 μg DNA using standard calcium phosphate precipitation, and 2 days later, cells were lysed in 200 μl lysis buffer (20 mM HEPES pH 7.4, 0.5% NP-40, 40 mM NaCl, 2 mM MgCl_2_, 1× protease inhibitor cocktail [Roche], 1× phosphatase inhibitor cocktail [Roche], 20 mM *N*-ethylmaleimide, 25 U ml^−1^ benzonase [Merck]). Lysates were incubated for 30 minutes on ice and cleared by centrifugation at 16,100 *g* for 10 minutes, and NaCl concentration was adjusted to 150 mM for coimmunoprecipitation to examine protein-protein interaction. Lysate was added to GFP-Trap_MA beads (ChromoTek), followed by rolling at 4°C for 2 hours. Beads were washed 4 times with wash buffer (20 mM HEPES, pH 7.4, 150 mM) and then resuspended in SDS loading buffer for analyses by immunoblotting.

### Protein assays.

For size exclusion, NSMCE1 and NSMCE3 were coexpressed in and purified from *E*. *coli* (23). The resulting complex was applied to a Superdex 200 Increase 5/150 GL column (GE Healthcare), preequilibrated in 20 mM HEPES.NaOH pH 7.5, 250 mM NaCl, 0.5 mM TCEP.

### Yeast 2-hybrid studies.

The NSMCE3 Pro209Leu and Leu264Phe mutations were compared with 2 previously characterized NSMCE3 mutants as controls (21, 25). Yeast 2-hybrid plasmids expressing NSMCE3, either WT or the mutated form as indicated, fused to the Gal4 DNA-binding domain or the empty vector control, and WT NSMCE1 or NSMCE4 fused to the Gal4-activation domain, were coexpressed in yeast cells, which were subsequently plated on the indicated media and grown at 30°C as described previously (22).

### Statistics.

All numerical data were analyzed using parametric statistical tests, namely Student’s *t* test, type 1, with 2 tails, and *P* values less than 0.05 were considered significant. All tests used and thresholds for significance are indicated in the figure legends.

### Study approval.

Informed consent was obtained from both participating families through studies approved by the Medical Ethical Committee of the University Medical Center Utrecht and the Institutional Review Board of Baylor College of Medicine. Parents of the children in family 1 provided written consent for the publication of photographs of the affected individuals.

## Author contributions

SNvdC, MPH, GMcG, DIR, SEP, JMM, and GvH conceived the study and wrote the manuscript. SNvdC, MPH, SCSN, IKC, JMvM, SWTL, PMvH, and SEP were involved in patient care and data collection. GMcG, DIR, OSW, MH, AA, LV, RH, SvL, MJvR, IR, KD, IJN, WPK, JSO, DAW, JJP, ARL, AWO, LHP, JMM, and GvH performed and coordinated experiments. DIR, SvL, MJvR, IR, IJN, and WPK performed bioinformatic analyses. EH performed genealogical analysis. OSW, MH, IKC, AA, LV, RH, JMvM, SWTL, SvL, MJvR, IR, KD, IJN, WPK, EH, JSO, PMvH, DAW, JJP, ARL, AWO, and LHP edited the manuscript.

## Supplementary Material

Supplemental data

## Figures and Tables

**Table 2:**
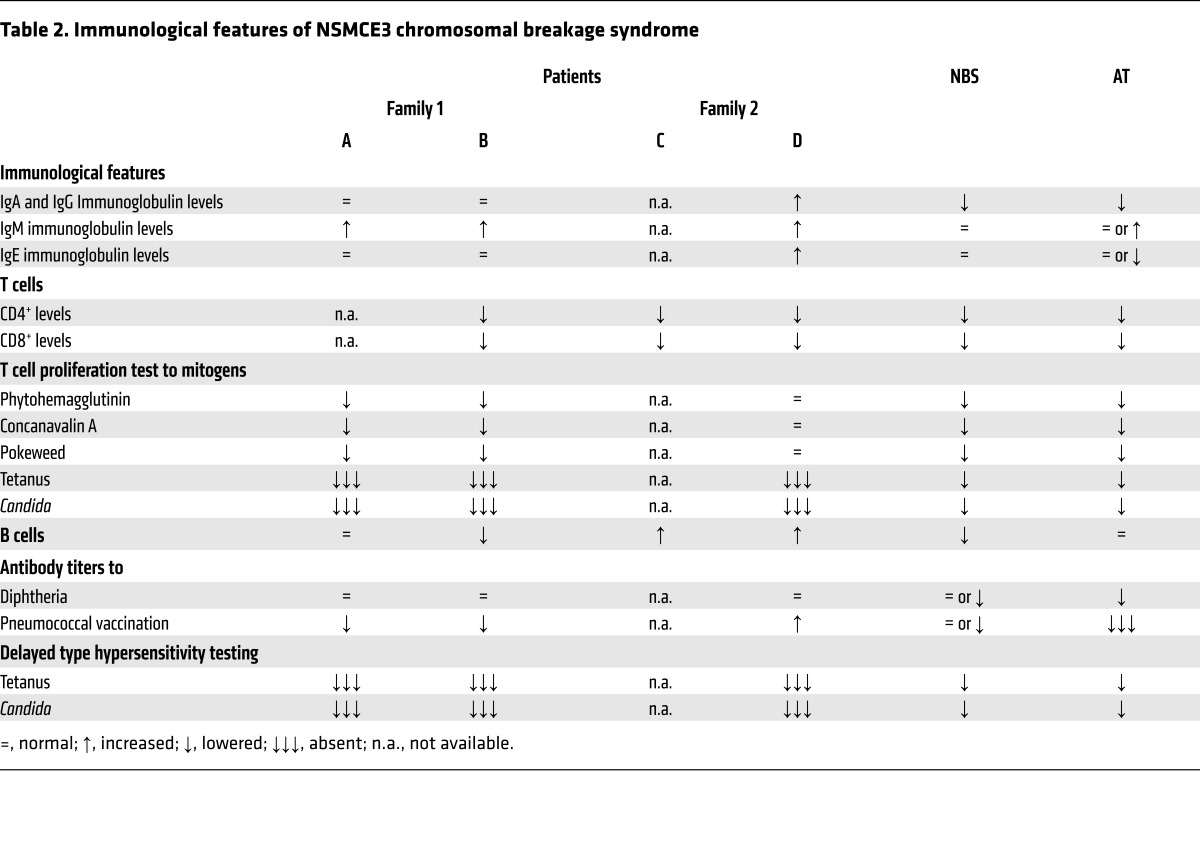
Immunological features of NSMCE3 chromosomal breakage syndrome

**Table 1:**
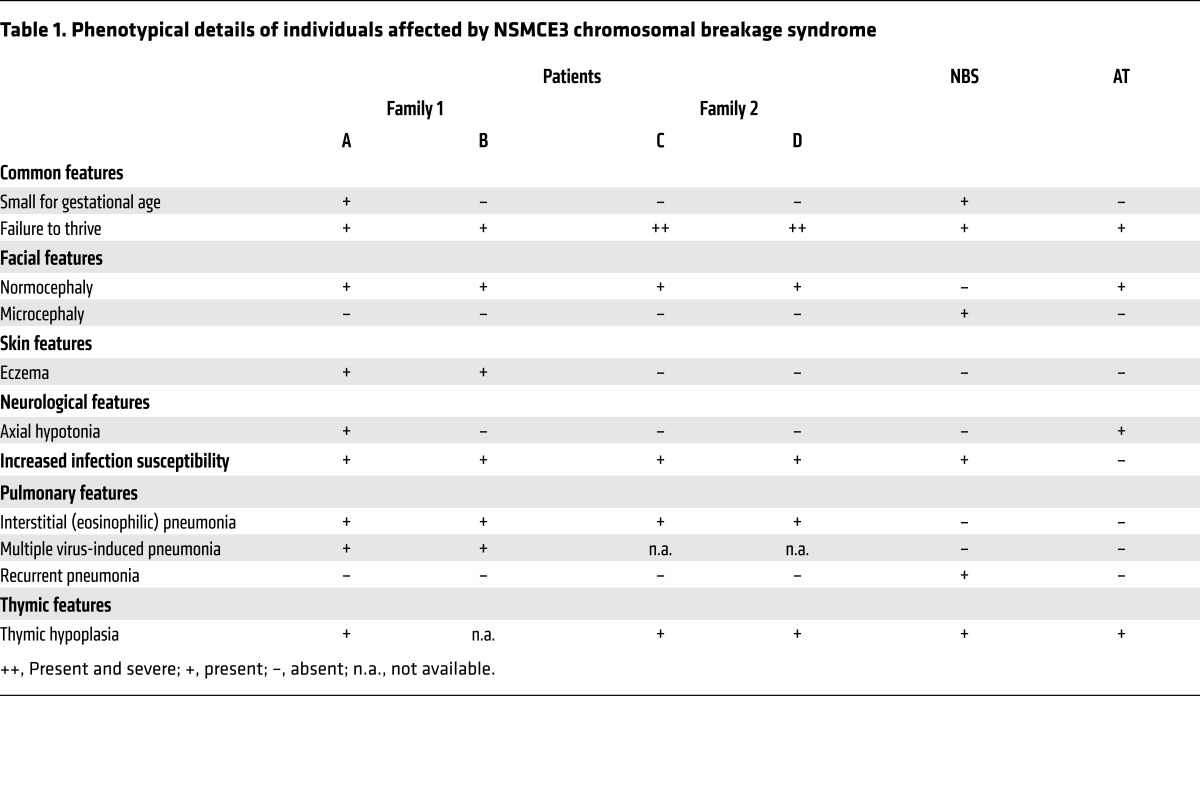
Phenotypical details of individuals affected by NSMCE3 chromosomal breakage syndrome
