# Supplementary material for: Destabilized SMC5/6 complex leads to chromosome breakage syndrome with severe lung disease
Source: J Clin Invest. 2016 Jul 18;126(8):2881–92. doi: 10.1172/JCI82890 (PMC4966312; doi:10.1172/JCI82890)
Supplement: Supplemental data [file JCI82890.sd.pdf]

Supplemental data to:

Biallelic missense mutations in *NSMCE3,* encoding a subunit of the SMC5/6 complex, cause a chromosome breakage syndrome with severe lung disease

Saskia N. van der Crabben[1,12], Marije P. Hennus[2,12], Grant McGregor[3,12], Deborah I. Ritter[4,12], Sandesh C.S. Nagamani[5,7], Owen S. Wells[3], Magdalena Harakalova[1], Ivan K Chinn[6-7], Aaron Alt[3], Lucie Vondrova[8], Ron Hochstenbach[1], Joris M. van Montfrans[9], Suzanne W. Terheggen-Lagro[10], Stef van Lieshout[1], Markus J. van Roosmalen[1], Ivo Renkens[1], Karen Duran[1], Isaac J. Nijman[1], Wigard P. Kloosterman[1], Eric Hennekam[1], Jordan S. Orange[6-7], Peter M. van Hasselt[11], David A. Wheeler[4-5], Jan J. Palecek[8], Alan R. Lehmann[3], Antony W. Oliver[3], Laurence H. Pearl[3], Sharon E Plon[4-7,12] Johanne M. Murray[3,12] and Gijs van Haaften[1,12].

[1]Department of Medical Genetics (Center for Molecular Medicine) University Medical Center Utrecht (UMCU), Utrecht, The Netherlands. [2]Pediatric Intensive Care, Wilhelmina Children's Hospital, University Medical Center Utrecht (UMCU), Utrecht, The Netherlands. [3]Genome Damage and Stability Centre, School of Life Sciences, University of Sussex, Falmer, Brighton, BN1 9RQ, United Kingdom. [4]Human Genome Sequencing Center, Baylor College of Medicine, Houston TX, USA. [5]Department of Molecular and Human Genetics, Baylor College of Medicine, Houston, TX, USA. [6]Department of Pediatrics, Baylor College of Medicine, Houston TX, USA. [7]Texas Children's Hospital, Houston TX, USA. [8]Central European Institute of Technology and Faculty of Science, Masaryk University, Brno, Czech Republic. [9]Pediatric Immunology and Infectious Diseases, Wilhelmina Children's Hospital, University Medical Center Utrecht (UMCU), Utrecht, The Netherlands. [10]Pediatric Pulmonary Diseases, Wilhelmina Children's Hospital, University Medical Center Utrecht (UMCU), Utrecht. [11]Metabolic Diseases, Wilhelmina Children's Hospital, University Medical Center Utrecht (UMCU), Utrecht, The Netherlands, [12]These authors contributed equally to this work.

Address correspondence to: Gijs van Haaften, Department of Medical Genetic, UMCU, STR. 1.305, PO Box 85060, 3508 AB, Utrecht, The Netherlands. Phone: 31.88.7567925; Email: g.vanhaaften@umcutrecht.nl or to: Johanne Murray, Genome Damage and Stability Centre, School of Life Sciences, University of Sussex, Falmer, Brighton, BN1 9RQ, United Kingdom. Phone: 44.1273. 877191; Email: j.m.murray@sussex.ac.uk

Authorship note: Saskia N. van der Crabben, Marije P. Hennus, Grant McGregor and Deborah I Ritter contributed equally to this work. Sharon E. Plon, Johanne M. Murray and Gijs van Haaften are equally contributing senior authors.

Conflict of interest statement: The authors declare the following:

Baylor College of Medicine (BCM) and Miraca Holdings Inc. have formed a joint venture with shared ownership and governance of the Baylor Miraca Genetics Laboratories which performs exome sequencing.

Dr. Plon is an employee of BCM and serves on the Scientific Advisory board of Baylor Miraca Genetic Laboratory.

Supplementary Figure 1. Aspecific cerebral calcifications in affected individuals A and B

Coronal CT-scan of the brain of the siblings of Family 1 showing aspecific cerebral calcifications. The arrows in coupes A-C (individual A) indicate the calcifications in the nuclei lentiformes and both frontal lobes. The arrows in coupe D (individual B) indicates the calcifications in the right frontal lobe.

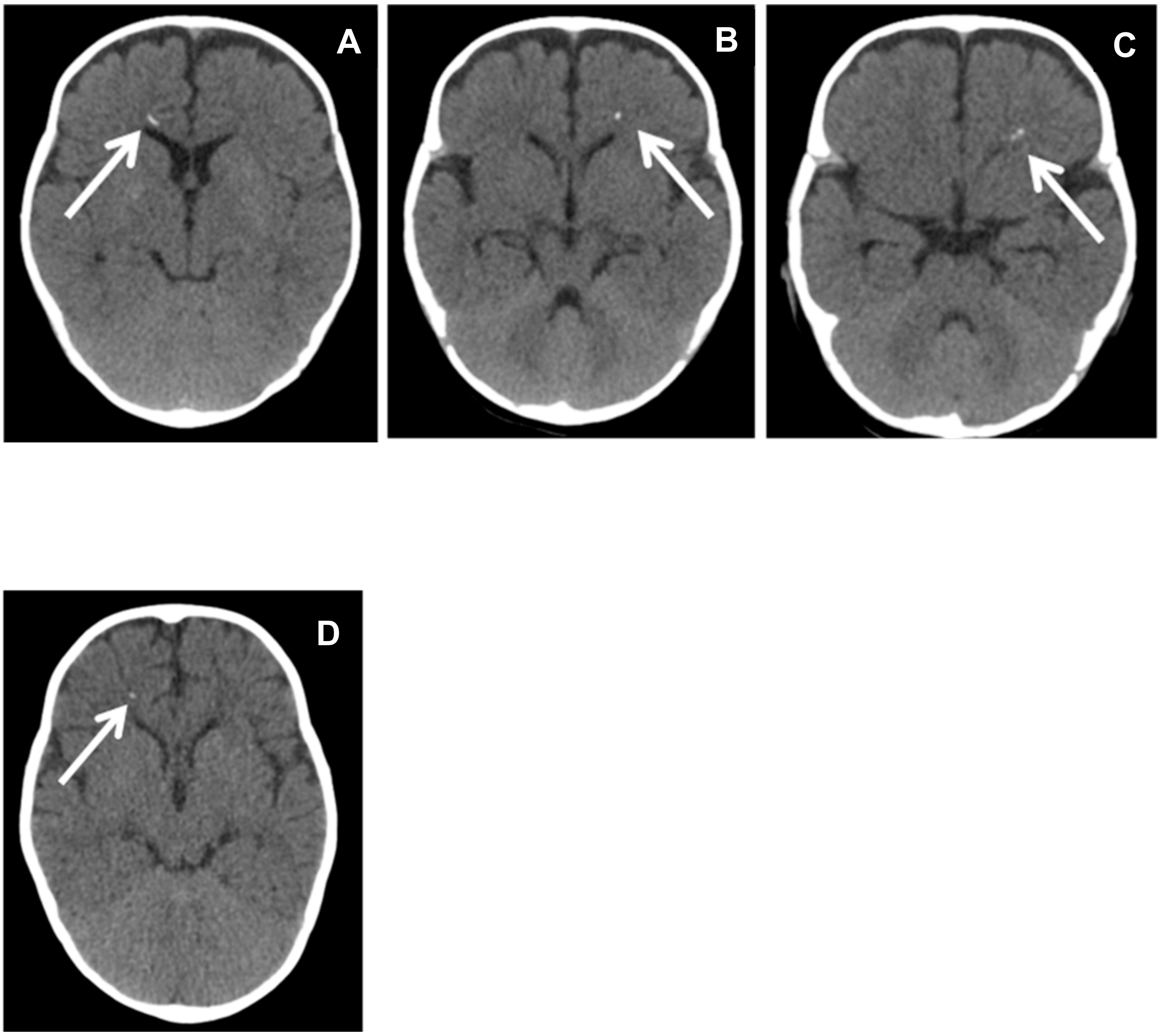

Supplementary Figure 2. Karyotypes of affected individuals A, B and D.

Individual A

Karyotyping of peripheral blood lymphocytes in individual A (age of 9 and 12 months (during her acute illness) respectively), showing the 5 metaphases, out of the 32 metaphases studied, that each contained one or two different *de novo* supernumerary marker chromosomes. Supernumerary markers are in the lower left of each karyotype and are indicated with “M”.

9 months

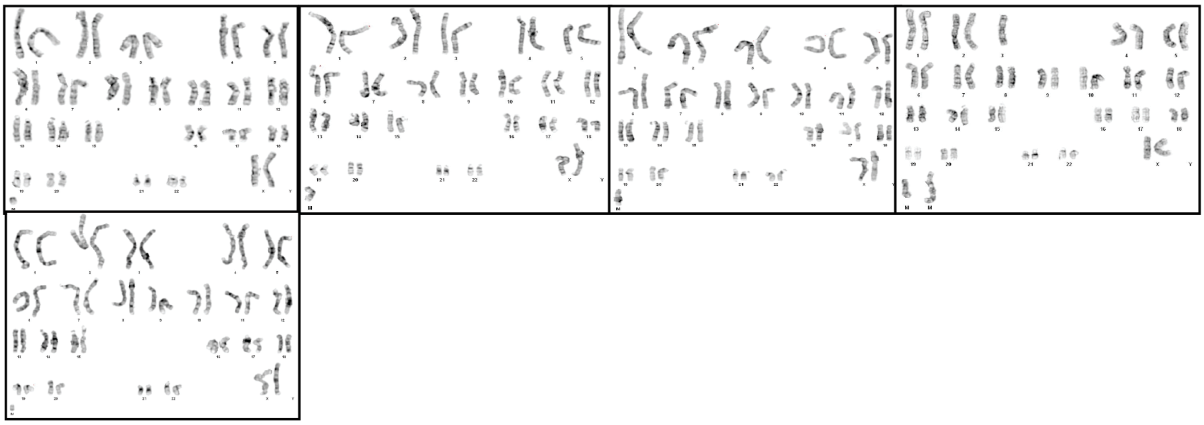

12 months

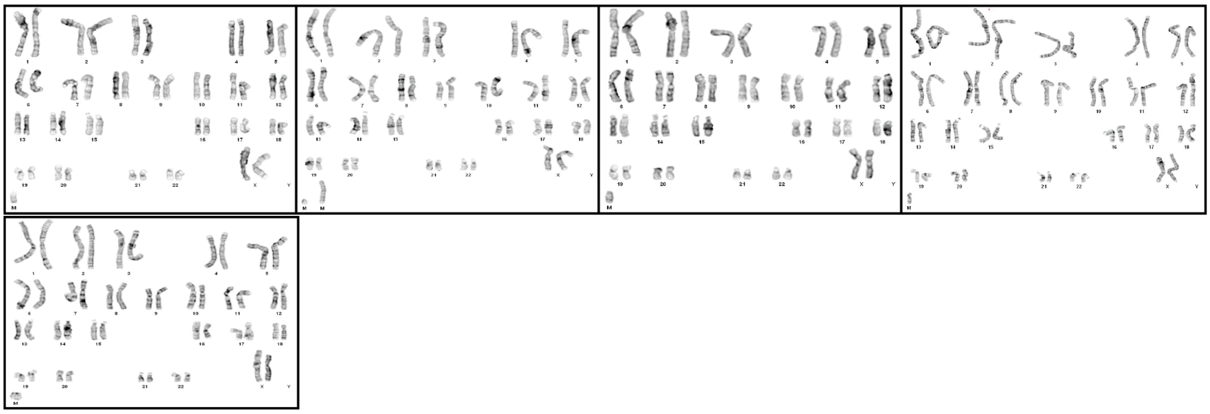

Individual B

In subject B, up to 8 different, *de novo* supernumerary markers were found in 12 out of the 50 metaphases investigated in lymphocytes. All markers seem to be unique, occurring in unique combinations in each aberrant cell; 3 of these also had a deleted or derivative chromosome. Three additional metaphases (bottom row) had a normal chromosome number with a terminal deletion affecting a different chromosome in each cell. From left to right: 47,XX,del(3)(q10),+mar; 46,XX,-14,+mar; 47,XX,+mar; 47,XX,del(4)(q22~24),+mar; 52,XX,-9,-9,+13,-20,+8mar; 49,XX,+3mar; 48,XX,-20,+3mar; 49,XX,+3mar; 49,XX,+3mar; 48,XX,der(12)t(5;12)(q13;p13),+2mar; 52,XX,-3,+7mar; 47,XX,+mar; 46,XX,del(7)(q10); 46,XX,del(11)(p13); 46,XX,del(21)(q22). Circles indicate *de novo* supernumerary chromosomes and the structural chromosome changes.

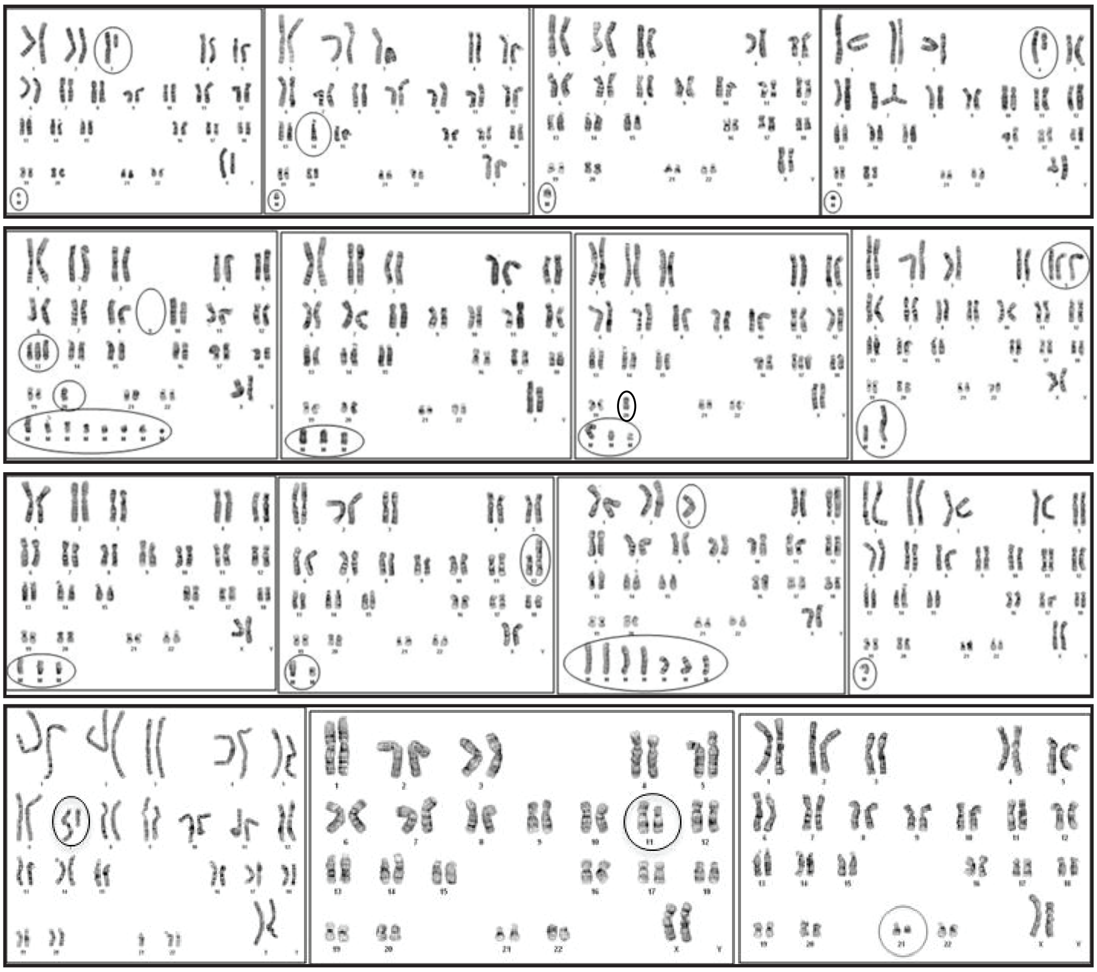

Individual D

100 metaphase spreads from early passage fibroblasts from individual D showed stochastic karyotypic abnormalities in 13 cells. Five of these cells showed an additional material of unknown chromosomal origin on chromosome 7 at band q32 and the remaining eight cells displayed random structural chromosomal changes. From left to right abnormal karyograms are: 46,XXadd(7)(q32) [4]; 47,XX,+X,add(1)(p36),add(7)(q32); 46,XX,del(1)(p32); 46,XX,del(5)(q13); 46,XX,del(10)(q?22); 45,XX,-4,add(15q26); 46,XX,tas(5;12)(q35;q24.3); 47,XX+mar; 47,XX+mar; 47,XX+mar

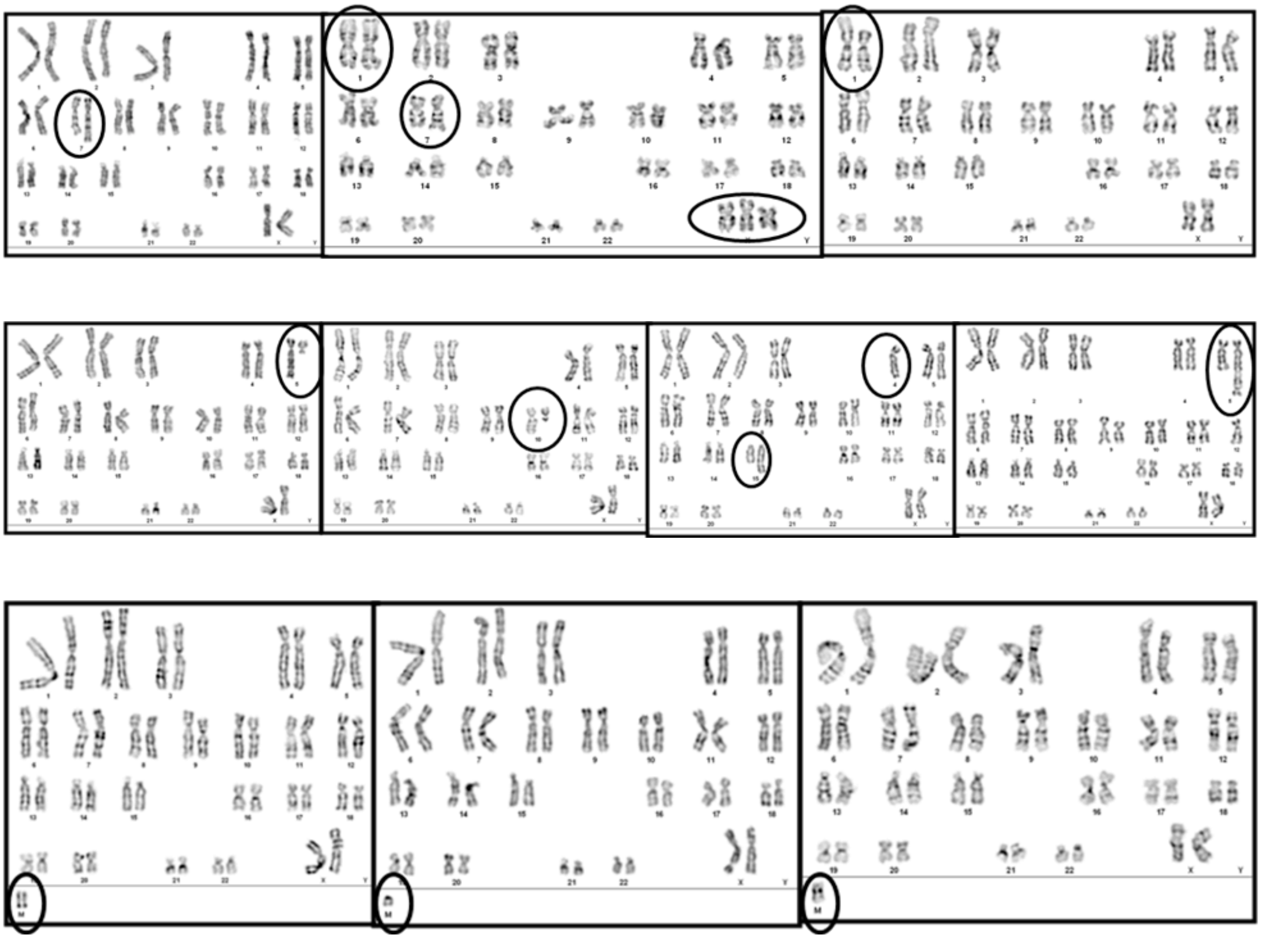

Supplementary Figure 3. Cell cycle profiles

FACS analysis of wild type fibroblasts (1BR) and patient B fibroblasts (GVH02) either untreated or after 18hrs in 250µM HU shows that the untreated patient fibroblasts have a similar cell cycle profile to wild type. After 18 hours in HU cells have accumulated in S phase to a similar extent in both cell lines. Left: FACS profiles; right: quantification of cell cycle profiles.

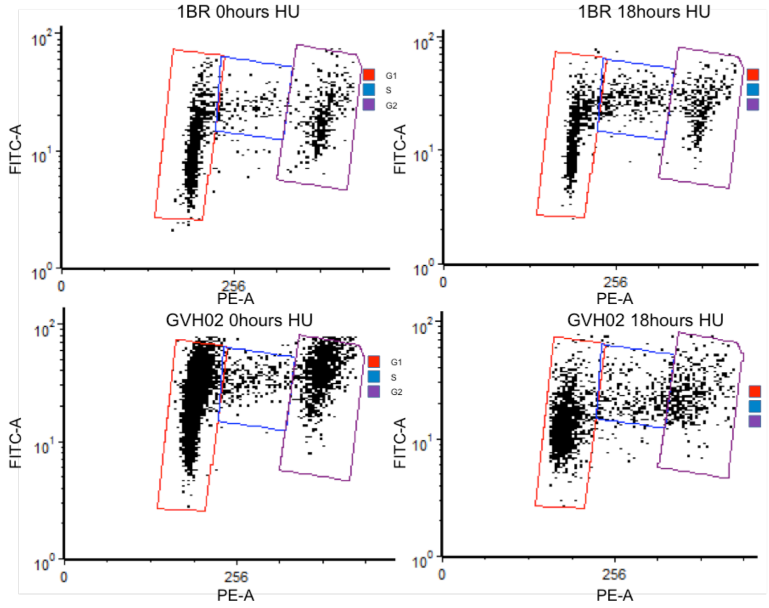

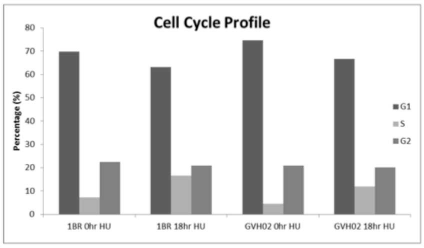

	G1 (%)	S (%)	G2 (%)
1BR 0hr HU	69.7	7.2	22.55
1BR 18hr HU	63.09	16.53	20.87
GVH02 0hr HU	74.61	4.48	20.95
GVH02 18hr HU	66.6	11.94	20.09

Supplementary Figure 4. Quantification of NSMCE3 levels in human U2OS cells

To determine the specificity of the NSMCE3 antibodies, NSMCE3 or an empty vector control were ectopically expressed in human U20S cells. In parallel NSMCE3 siRNA knock down or a scramble control was performed in U2OS cells. Immunoblots were probed with anti-SMC5 and anti-NSMCE3. Tubulin was used as a loading control. Left panel: Recombinant NSMCE3 is detectable as a strong 32kD band. Right panel: Endogenous NSMCE3, which also runs at 32kD, is reduced after NSMCE3 siRNA knockdown compared to the control (higher exposure than on left). In addition SMC5 levels were slightly reduced in the siNSMCE3 suggesting that knockdown of NSMCE3 destabilises the SMC5/6 complex similarly to the cells from the affected individuals.

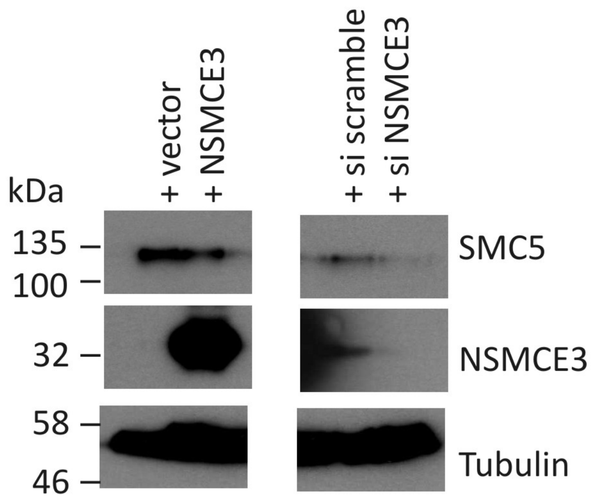

Supplementary data

Family 1

Individual A was born at term (38 weeks gestational age), small for gestational age (birth weight 2420 gram (<2,5 SD)). Physical examination at time of clinical evaluation for failure to thrive and axial hypotonia, revealed mild dysmorphic features (a large open fontanel, thin, translucent skin, prominent forehead veins) as well as eczema. Coronal CT-scan of the brain identified aspecific cerebral calcifications (Supplementary Figure 1). Clinical investigations performed for reasons of eczema and food allergies revealed high titres of total IgE class antibodies (473 kU / L) including specific antibodies against several dietary antigens.

After admission for respiratory failure, PCR-analysis of bronchoalveolar lavage fluid (BALF) was positive for human metapneumovirus, rhinovirus, cytomegalovirus and human herpes virus type-6. Peripheral blood showed normal numbers for leukocytes including differentiation. Serum IgM, IgA and IgG levels were within normal ranges for age (0.92, 0.75 and 7.0 g/L, respectively). Specific antibodies against Pneumococcus and *Haemophilus Influenzae* were absent despite recent booster vaccinations. Lymphocyte proliferation tests showed suboptimal proliferation upon stimulation with mitogens (including Concanavalin A and Pokeweed Mitogen) and an absence of proliferation upon stimulation with recall antigens (including tetanus toxoid, diphtheria and candida) despite recent vaccinations with tetanus toxoid and diphtheria.

Lymphocyte-karyotyping showed one or two *de novo* supernumerary marker chromosomes that were unique to each 16% of cells. Based on G-banding pattern it was not possible to identify the chromosomal content of the markers; BAC-based array-CGH showed a normal female profile. Karyotyping of skin fibroblasts showed a normal female (46,XX) karyotype in all 32 metaphases investigated. Sensitivity to UV radiation tested by routine clinical diagnostics in fibroblasts showed a return to a normal level of DNA-synthesis, thereby excluding Cockayne syndrome and xeroderma pigmentosum. DNA-synthesis in the

fibroblasts of individual A after the use of gamma-radiation decreased which ruled out the possibility of ataxia telangiectasia and Nijmegen breakage syndrome. Methylation-specific MLPA on DNA of individual A to rule out a maternal UPD 7 or 14 was normal.

Post-mortem examination in individual A revealed diffuse bilateral alveolar damage as well as a small thymus with diffuse B-cell infiltrates.

Individual B was born at term after an uncomplicated pregnancy. Upon admission for pneumonia at the age of 14 months, PCR-analysis of BALF was positive for Respiratory Syncytial Virus and *Mycoplasma Pneumoniae*. Coronal CT-scan of the brain identified aspecific cerebral calcifications (Supplementary Figure 1). Serum levels of IgM, IgA and IgG were within normal ranges (0.93, 1.0 and 7.48 g/L, respectively). Specific antibodies against pneumococcal antigens were relatively low despite recent vaccinations with 7-valent Pneumococcal conjugate vaccine (Prevenar). Lymphocyte phenotyping showed low numbers of CD4+ and CD8+ T cells (537 and 248 cells/microliter, respectively), with normal percentages of naïve T cells. B cell and NK cell numbers were normal (618 and 214 x 10E9 / L). Lymphocyte proliferation tests showed normal proliferation upon stimulation with mitogens (including Concanavalin A and Pokeweed mitogen), but an absence of proliferation upon stimulation with recall antigens (including tetanus toxoid, diphteria and candida) despite recent vaccinations with tetanus toxoid and diptheria.

Additional analysis on a head hair of individual B showed a normal cysteine-content thereby excluding trichothiodystrophy. Lymphocyte-karyotyping of individual B revealed in about 30% of cells a similar pattern of unique combinations of up to 8 *de novo*, supernumerary marker chromosomes per cell with or without additional chromosomal rearrangements. Array-CGH showed a normal female (46,XX) profile. Karyotyping of skin fibroblasts showed a normal female (46,XX) karyotype in all 32 metaphases investigated. Additional genetic screening (sequencing and MLPA) in DNA of subject B for the *TTF1* gene was normal. Mitomycin C testing of fibroblasts of individual B to exclude Fanconi's anemia did not show an abnormally

increased chromosome aberration rate, but cells grew very poorly and only few were available for analysis.

Family 2

Individual C was born at full gestation via normal vaginal delivery. The birth weight of 2.5 kg (Z-score of -1.5) and birth length of 45.72 cm (Z-score of -1.5) were within normal limits. He was clinically evaluated at the age of five months for failure to thrive. Physical examination revealed mild dysmorphic features (a large anterior fontanel, wide spaced eyes, flat mid-face, and flattened nasal bridge). A review of skeletal survey did not reveal any features that would be suggestive of skeletal dysplasia. Alkaline phosphatase levels were normal and there was no evidence for metabolic bone disease. A karyotype analysis showed normal male chromosome constitution, 46, XY. Analysis of urine amino acids, plasma amino acids were unremarkable. A urine organic acid analysis revealed the presence of 3-methyl-glutaconic acid. Urinary mucopolysaccaride levels were normal. A cause for the growth restriction was not apparent. No specific clinical diagnosis was determined. At 14 months of age he was hospitalized for pneumonia and was treated with antibiotics. Despite broad antimicrobial treatment, his respiratory status steadily declined, and he was placed on mechanical ventilation.

Bronchoscopy revealed findings suggestive of eosinophilic pneumonia. Lung damage progressed diffusely, similar to the siblings in Family 1, and he could not be adequately ventilated. He expired at 15 months of age. Post-mortem examination demonstrated eosinophilic pneumonia, bronchiolitis obliterans, and organized pneumonia. Lymphocyte phenotyping at 14 months of age showed low numbers of $CD3^+$, $CD4^+$ and $CD8^+$ T cells (567, 397, and 223 cells/microliter, respectively). Total T cells represented 34% of lymphocytes; this decreased percentage remained consistent when testing was repeated at 15 months of age. B cell numbers were slightly elevated, and NK cell numbers were slightly diminished (968 and 37 cells/microliter, respectively).

Individual D was born at 38 weeks of gestation. Her birth weight was 2.63 kg (Z-score of -1.5) and birth length was 45.72 cm (Z-score -1.5). At around 2-3 months, she started falling off from her usual growth curve and subsequently developed significant restriction of growth. Her height and weight were always well below the 5th percentile. At 5 months of age, she was treated with antibiotics for pneumonia. At the age of 13 months, she was hospitalized for pneumonia after initial antimicrobial treatment following otitis media. Her respiratory status progressively declined, requiring intubation and mechanical ventilation.

PCR-analysis of a tracheal aspirate revealed the presence of adenovirus. Despite high dose steroids and antimicrobial therapies, the pulmonary disease continued to progress, and the patient underwent cadaveric lung transplantation at 15 months of age. During the surgical procedure, a small atrophic thymus was noted. After a complicated post-transplantation course, the patient recovered and was discharged home.

From the age of 28 months onward, she developed sequential infections: osteomyelitis of the sternum, oral thrush and consecutive central line infections and bacteremia. At 28 months of age, she had *Candida albicans* osteomyelitis of the sternum that required treatment with intravenous amphotericin. She also developed a central line infection from *Serratia marcescens.* A month later, she was treated for oral thrush. Shortly afterwards, she was admitted to the hospital for pseudomonal infection of the central line and bacteremia. During hospitalization, she then developed central line infections from *E. coli*, *E. faecalis*, and coagulase-negative staphylococci. Ultimately all immune suppression was discontinued. Nonetheless, her clinical status deteriorated, and the patient died at 32 months of age. Autopsy confirmed absence of the thymus.

Immunoglobulin levels obtained at 9 and 12 months of age demonstrated normal serum levels of IgM, IgA and IgG (1.22, 0.64, 8.48 and 0.58, 0.44, 8.13 g/L, respectively) and increased levels of IgE (0.48 and 1.06 g/L, respectively). She was formally evaluated by

Allergy and Immunology service at 12 months of age and was noted to have had no history of infections (aside from pneumonia at 5 months of age) and no record of recurrent need for antibiotics. Specific antibody titers against *corynebactierium diphtheria*, *clostridium tetani*, and *H. influenzae* were normal. Furthermore, she had protective antibody titers to 6 of 6 protein-conjugated pneumococcal serotypes and 1 of 6 pneumococcal serotypes not found in the Prevnar 7 immunizations that she had received. The CH50 was normal. Delayed type hypersensitivity testing, however, demonstrated no significant responses to *Candida* or tetanus. Upon hospitalization at the age of 13 months, further immunologic testing demonstrated diminished numbers of $CD3^+$, $CD4^+$, and $CD8^+$ cells (1,053, 768, and 263 cells/mm^3 respectively) and an increased number of B cells (842 cells/mm^3). Similar to her affected brother (individual C), the total T cell percentage was low (47% of lymphocytes). The NK cell percentage was normal (14%, absolute number 304 cells/mm^3). T cell proliferation studies showed excellent responses to mitogens (phytohemagglutinin, concanavalin A, and pokeweed mitogen. She had no proliferative responses to tetanus or *Candida*. A serum tumor necrosis factor alpha level was also obtained and was elevated (11.8 pg/mL, normal less than 8.2 pg/mL). At 31 months of age, while receiving immunosuppression, repeat immune phenotyping showed marked lymphopenia (absolute count of 589 cells/mm^3) with low percentages of T and B cells (37% and 22%, respectively) and a compensatory increase in NK cell percentage (34%)

Results from clinical radiosensitivity on **lymphoblastoid cell lines** showed the presence of significant radiosensitivity (16% survival). Immunoblotting showed reduced levels of ATM protein (48% of normal; 31% of normal on second blot). This did not support a diagnosis of conventional A-T as patient with Ataxia Telangiectasia with trace amounts of protein never show levels greater than 15%. As occasional patients with A-T have been described with near normal ATM protein levels with poor kinase activity, a kinase assay using SMC1 and nibrin as downstream substrates was done. SMC1 phosphorylation was reduced; nibrin phosphorylation was absent. Sequencing of the AT gene revealed no mutations.
